# Long-term drug modification to the surface of mesenchymal stem cells by the avidin-biotin complex method

**DOI:** 10.1038/s41598-017-17166-8

**Published:** 2017-12-05

**Authors:** Yukiya Takayama, Kosuke Kusamori, Mika Hayashi, Noriko Tanabe, Satoru Matsuura, Mari Tsujimura, Hidemasa Katsumi, Toshiyasu Sakane, Makiya Nishikawa, Akira Yamamoto

**Affiliations:** 1 Department of Biopharmaceutics, Kyoto Pharmaceutical University, Yamashina-ku, Kyoto, 607-8414 Japan; 2 Laboratory of Biopharmaceutics, Faculty of Pharmaceutical Sciences, Tokyo University of Science, 2641 Yamazaki, Noda, Chiba, 278-8510 Japan

## Abstract

Mesenchymal stem cells (MSCs) have various functions, making a significant contribution to tissue repair. On the other hand, the viability and function of MSCs are not lasting after an *in vivo* transplant, and the therapeutic effects of MSCs are limited. Although various chemical modification methods have been applied to MSCs to improve their viability and function, most of conventional drug modification methods are short-term and unstable and cause cytotoxicity. In this study, we developed a method for long-term drug modification to C3H10T1/2 cells, murine mesenchymal stem cells, without any damage, using the avidin-biotin complex method (ABC method). The modification of NanoLuc luciferase (Nluc), a reporter protein, to C3H10T1/2 cells by the ABC method lasted for at least 14 days *in vitro* without major effects on the cellular characteristics (cell viability, cell proliferation, migration ability, and differentiation ability). Moreover, *in vivo*, the surface Nluc modification to C3H10T1/2 cells by the ABC method lasted for at least 7 days. Therefore, these results indicate that the ABC method may be useful for long-term surface modification of drugs and for effective MSC-based therapy.

## Introduction

Mesenchymal stem cells (MSCs) are generally isolated from a variety of tissues including bone marrow, adipose tissue, umbilical cord blood, and placenta^1–3^. Unlike other types of stem cells (e.g., embryonic stem cells or induced pluripotent stem cells), MSCs are easily prepared without ethical concerns and hardly pose a risk of teratoma formation after a transplant^4^. Moreover, the self-renewal ability and proliferation of MSCs enable mass production of these cells for basic researches and clinical trials. Until now, many studies have shown that MSCs have a variety of unique characteristics such as secretion of cytokines and growth factors, a tumour-homing ability, and multipotency^5–8^. Bone marrow- or adipose tissue-derived MSCs have been widely used as therapeutic cells and the amount of cytokines secreted by MSCs varies depending on the isolation site in the body^9^. These functions of MSCs therefore can provide some benefits for tissue repair, and the therapeutic potential of MSCs for a cell-based therapy against various diseases has already been demonstrated in animals and humans^10,11^.

However, the function and viability of MSCs are generally transient and low after an *in vivo* transplant. This is because most of transplanted MSCs may disappear under the influence of immune cells and via negative effects of endogenous or environmental changes (inflammation, ischemia-reperfusion, and the lack of nutrition and oxygen)^12,13^. To overcome these disadvantages of MSC transplantation, recent studies have shown that genetic engineering or surface chemical modification improves and diversifies the therapeutic potential of MSCs^12,14,15^. These methods can not only improve a cellular function but also impart a completely different function to MSCs. Although genetic engineering methods are frequently applied to various cells and the engineered MSCs may be effective in the treatment of various diseases^16–18^, some disadvantages remain: 1) low transfection efficacy, 2) lengthy cultivation for the establishment of a stable gene-expressing clone, and 3) risks associated with viral vectors. On the other hand, chemical modification methods (cell surface modification methods), including the covalent bond method, the electrostatic interaction method, and the hydrophobic bond method, can overcome these disadvantages of genetic engineering methods^15,19^ because these methods offer rapidity of the chemical modification and high efficacy. However, the instability (transient nature) of surface modification of cells is a major problem in this approach^20^. A method for long-term drug modification to cells with ease and safety is therefore highly desirable for functionalisation of MSCs.

Avidin (or streptavidin) and biotin are known to form a firm non-covalent bond, and this non-covalent bond is one of the strongest in nature^21^. The binding of avidin to biotin is very fast and irreversible with high specificity and has been applied to the detection or recovery of peptides, proteins, and nucleic acids, and for chemical modification of various molecules^22,23^, which is called the avidin-biotin complex method (ABC method). That is, the ABC method may overcome the disadvantages of conventional methods for drug modification of cells owing to the stability of the bond and rapidity of the reaction. Although some researchers have reported application of the ABC method to cells^24–26^, the duration of surface modification of cells and the influence of the ABC method on cells have hardly been evaluated. Because MSCs have unique characteristics such as the differentiation ability and homing ability, the influence of the ABC method on these characteristics should be examined for practical application of MSC-based therapy.

In this study, we evaluated the *in vitro* and *in vivo* duration of surface modification of MSCs and the influence of the ABC method on characteristics of MSCs. To evaluate the surface modification of MSCs, we selected the murine mesenchymal stem cells, C3H10T1/2 cell line, and two reporter proteins to be modified: NanoLuc luciferase (Nluc) and enhanced green fluorescent protein (GFP). First, we examined the drug modification to the surface of C3H10T1/2 cells with fluorescently labelled streptavidin or with biotin-GFP by the ABC method. Then, the cell viability was evaluated using biotinylation reagents at various concentrations and the magnitude of Nluc modification of C3H10T1/2 cells was optimised. Moreover, the *in vitro* duration of Nluc modification of C3H10T1/2 cells was evaluated using the optimised Nluc modification procedure. On the other hand, cell proliferation, cell attachment, migration ability and differentiation ability of C3H10T1/2 cells were evaluated to assess possible adverse effects of Nluc modification by the ABC method. To evaluate the efficacy of surface modification by the ABC method, GFP-modified C3H10T1/2 cells were analysed on a flow cytometer. Finally, the *in vivo* duration of surface modification of C3H10T1/2 cells was evaluated in nude mice by means of an *in vivo* imaging system.

## Results

### Drug modification of the surface of cells

**Figure 1:**
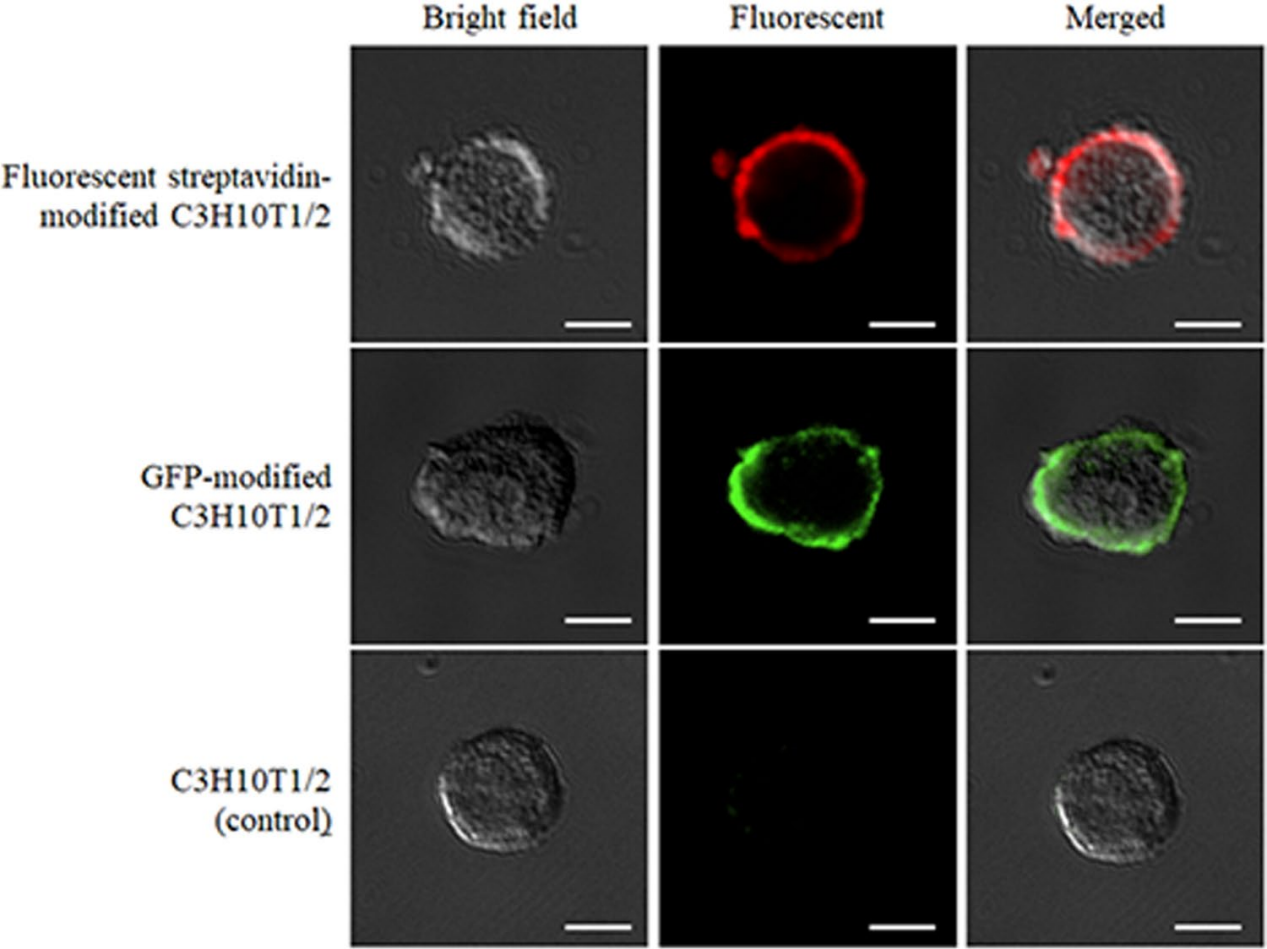
The typical images of surface-modified C3H10T1/2 cells. Fluorescent streptavidin-modified C3H10T1/2 cells were prepared by adding Alexa Fluor 647 conjugated streptavidin to biotinylated C3H10T1/2 cells. GFP-modified C3H10T1/2 cells were prepared by adding biotin-GFP to avidinated C3H10T1/2 cells. C3H10T1/2 cells (control) were prepared by adding biotin-GFP to unmodified C3H10T1/2 cells after addition of avidin. Scale bars represent 20 μm.

Figure 1 shows the fluorescent streptavidin-modified C3H10T1/2 cells, the GFP modified C3H10T1/2 cells and C3H10T1/2 cells (control), respectively. C3H10T1/2 cells (control) were prepared by adding biotin-GFP to unmodified C3H10T1/2 cells after addition of avidin. The strong fluorescence was observed only on the surface of fluorescent streptavidin-modified C3H10T1/2 cells and GFP-modified C3H10T1/2 cells, whereas slight signals were observed in C3H10T1/2 cells (control).

### Optimisation of biotinylation of cells and of surface modification

**Figure 2:**
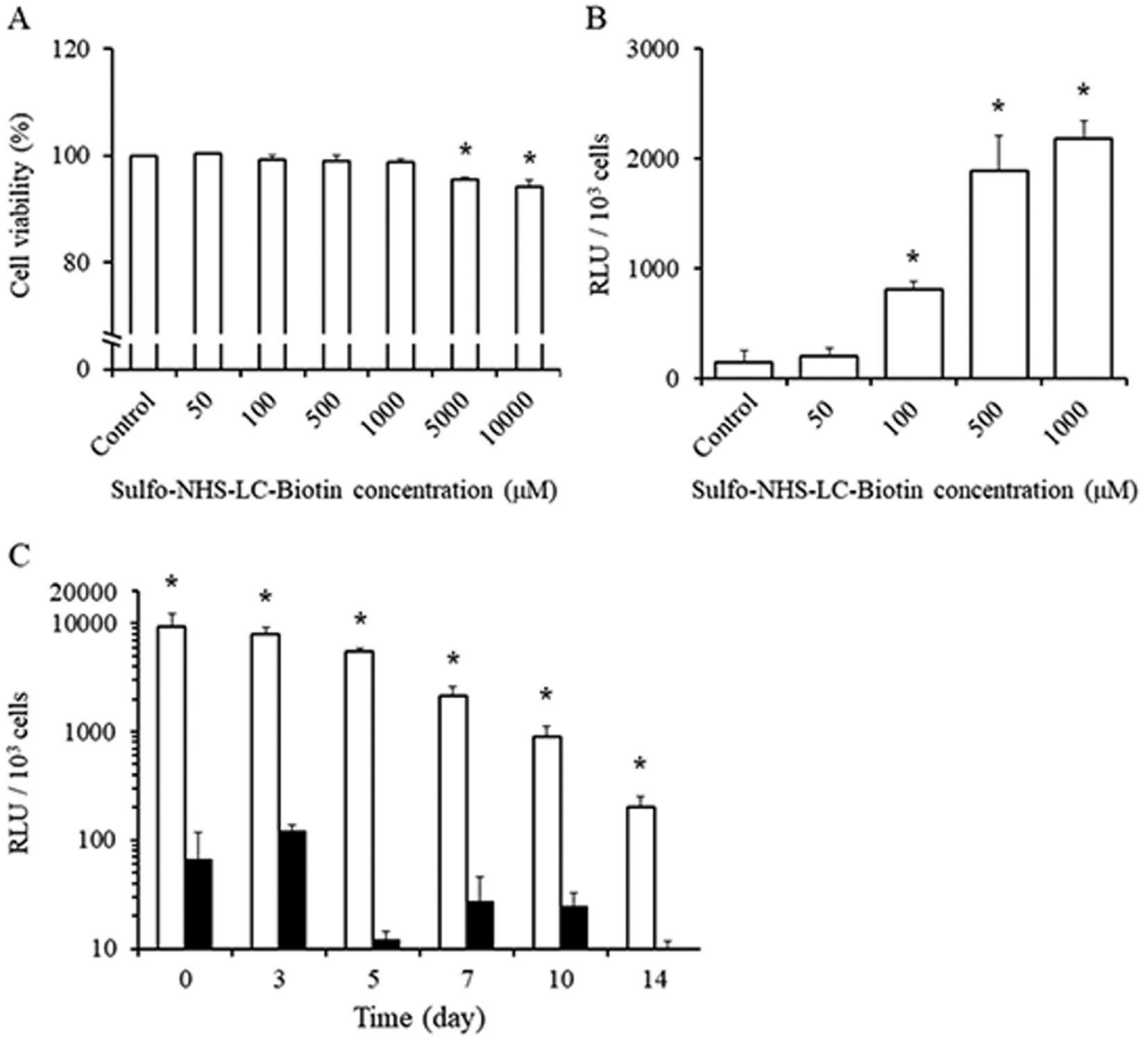
Optimisation of surface modification of C3H10T1/2 cells. (**A**) Cell viability of C3H10T1/2 cells biotinylated using sulfo-NHS-LC-biotin at various concentrations (50, 100, 500, 1000, 5000, or 10000 μM). (**B**) The relative luminescence unit (RLU) of Nluc-modified C3H10T1/2 cells biotinylated with sulfo-NHS-LC-biotin at various concentrations (50, 100, 500, or 1000 μM). (**C**) The RLU of (white) Nluc-modified C3H10T1/2 cells and (black; control) C3H10T1/2 cells incubated with Nluc. These experiments were conducted in duplicate, and results of a representative experiment are shown. Results are expressed as mean ± SD of three to four experiments. *p < 0.05 a statistically significant difference compared with the control group.

We then examined the optimal concentration of sulfo-NHS-LC-biotin. The viability of cells was hardly affected by 1000 μM or lower sulfo-NHS-LC-biotin (Fig. 2A). Then, 500 and 1000 μM sulfo-NHS-LC-biotin showed the sufficient Nluc modification to cells (Fig. 2B). Furthermore, 1000 μM sulfo-NHS-LC-biotin showed the higher Nluc modification to cells than 500 μM sulfo-NHS-LC-biotin. Therefore, 1000 μM sulfo-NHS-LC-biotin was used for the modification in the following experiments. Figure 2C shows the duration of Nluc modification of the cells by the ABC method. Nluc modification of C3H10T1/2 cells lasted for at least 14 days and the amount of Nluc modified to cells remained at more than 80% for 5 days. In contrast, the amount of Nluc of C3H10T1/2 cells simply incubated with Nluc was hardly detectable. Furthermore, the fluorescence of C3H10T1/2 cells modified with NHS-FITC decreased to less than 10% within 3 days after modification and disappeared completely thereafter as previously reported (Supplementary Fig. 1).

### Characteristics of surface-modified cells

**Figure 3:**
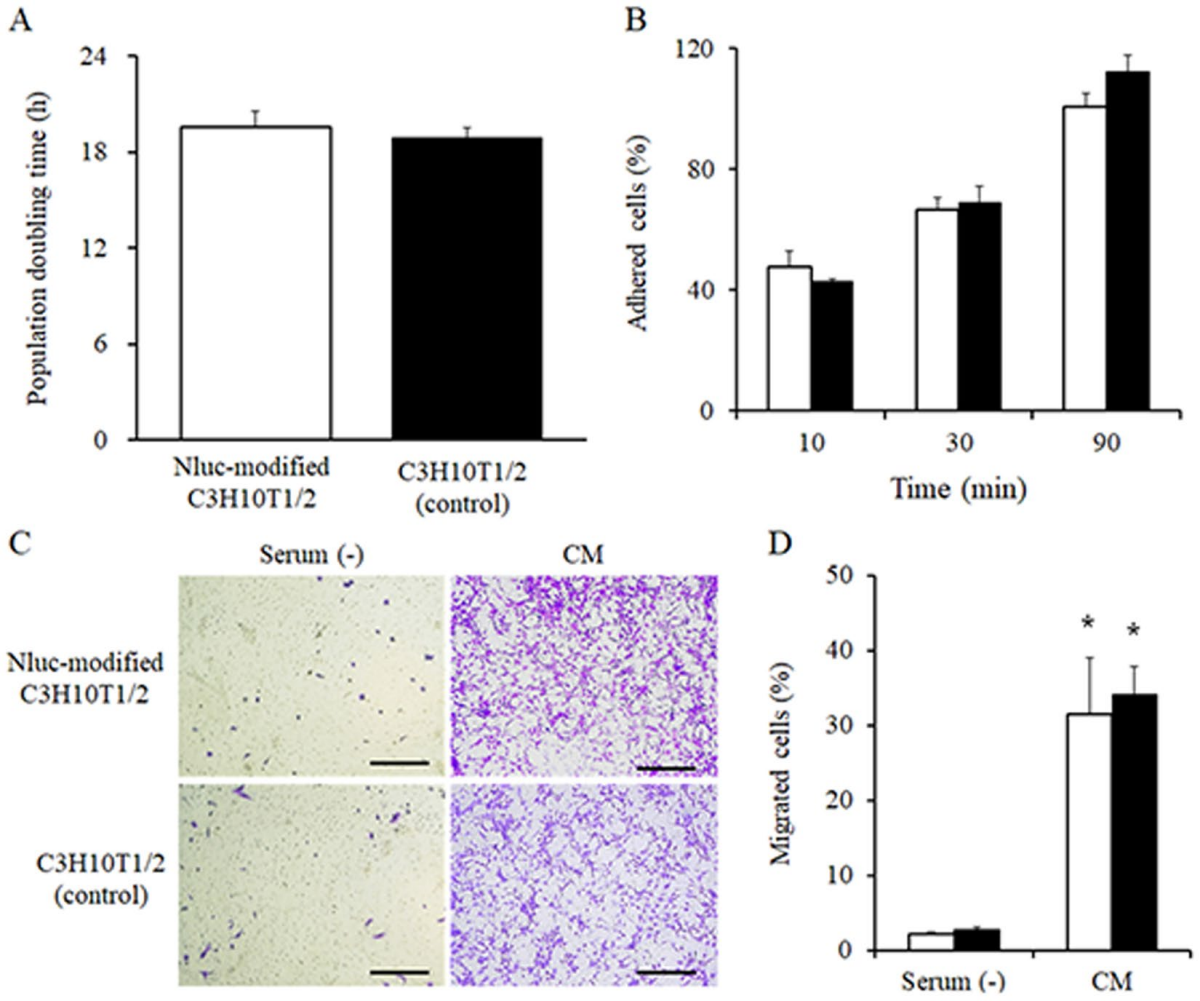
Characteristics of Nluc-modified C3H10T1/2 cells. (**A**) Population doubling time (PDT) of the cells. (**B**) The percentage of cells attached to a cell culture plate after 10, 30, and 90 min. (**C**) The typical images and (**D**) the number of migrating cells in Transwell culture inserts in the serum-free medium (Serum (−)) or the conditioned medium (CM). (White) Nluc-modified C3H10T1/2 cells and (black) C3H10T1/2 cells (control). *p < 0.05 a statistically significant difference compared with the Serum (−) group. These experiments were conducted in duplicate, and a representative experiment is shown. Results are expressed as mean ± SD of three to four experiments.

**Figure 4:**
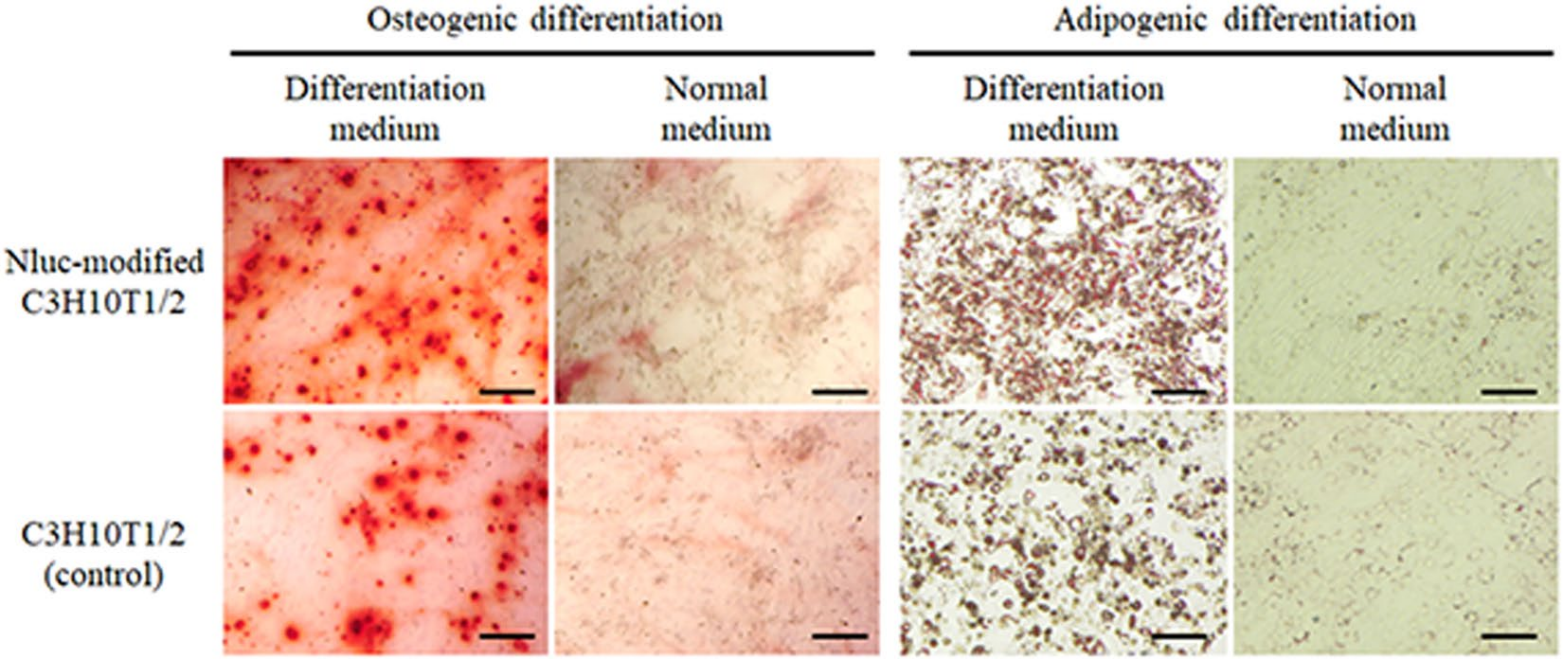
Differentiation of Nluc-modified C3H10T1/2 cells. The typical images of Nluc-modified C3H10T1/2 cells and C3H10T1/2 cells (control) that differentiated into osteoblasts or adipocytes. Scale bars represent 200 μm.

Figure 3 shows the population doubling time (PDT) as an indicator of cell proliferation, the attachment to the culture plate, and the migration ability of Nluc-modified C3H10T1/2 cells. The PDT of Nluc-modified C3H10T1/2 cells and C3H10T1/2 cells (control) was 19.6 and 18.9, respectively (Fig. 3A). Then, the proportion of cells attached to culture plates among Nluc-modified C3H10T1/2 cells and C3H10T1/2 cells (control) was 48% and 43% after 10 min, 67% and 69% after 30 min, and 101% and 113% after 90 min, respectively (Fig. 3B). In addition, no significant differences in cell attachment at 24 h between Nluc-modified C3H10T1/2 cells and C3H10T1/2 cells (control) were observed (Supplementary Fig. 2). The percentage of migrant Nluc-modified C3H10T1/2 cells and migrant C3H10T1/2 cells (control) increased to 31.4% and 34.0%, respectively, in the conditioned medium from colon26 cells as compared with the serum-free medium (2.1% and 2.8%, respectively; Fig. 3C,D). According to these results, Nluc-modified C3H10T1/2 cells showed almost the same characteristics as did the C3H10T1/2 cells (control). Moreover, Fig. 4 shows the images of cells differentiated into osteoblasts and adipocytes. Nluc-modified C3H10T1/2 cells and C3H10T1/2 cells (control) similarly differentiated into osteoblasts and adipocytes in the differentiation media. In addition, Nluc-modified cells and untreated cells also differentiated into chondrocytes (Supplementary Fig. 3). On the other hand, both groups of cells did not differentiate into any types of cells in a normal medium (DMEM supplemented with 15% of heat-inactivated fetal bovine serum and 0.6% of an antibiotic-antimycotic mixed solution). These results showed that the surface modification by the ABC method hardly affected the characteristics of MSCs.

### Efficacy of surface modification of the cells

**Figure 5:**
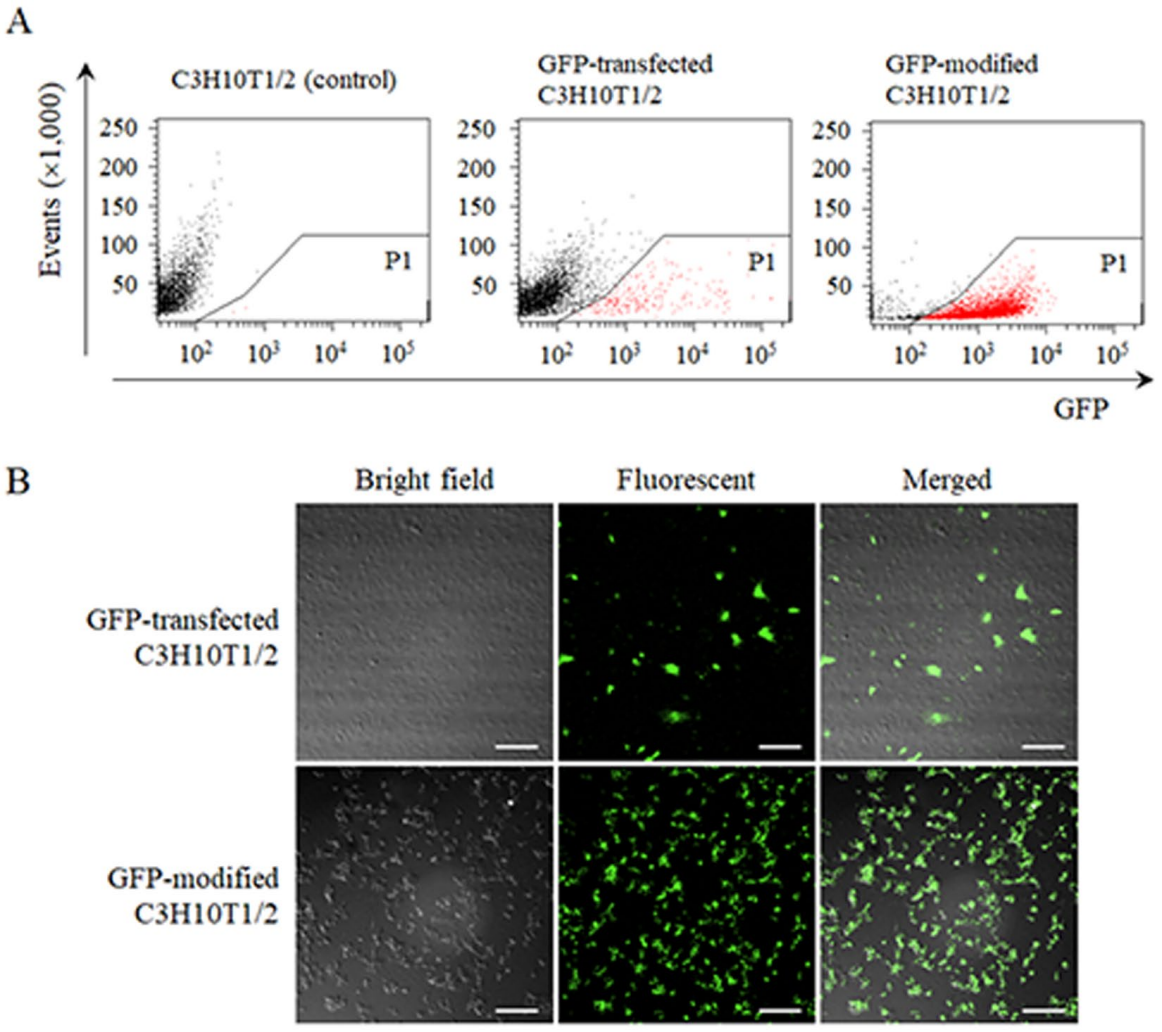
The surface modification efficacy of GFP-modified C3H10T1/2 cells. (**A**) Flow cytometric analysis of GFP-positive cells. GFP-modified C3H10T1/2 cells and GFP-transfected C3H10T1/2 cells were analysed by flow cytometry. Region P1 (red dots) in the plot shows GFP-positive cells. (**B**) The typical images of GFP-modified C3H10T1/2 cells and GFP-transfected C3H10T1/2 cells. Scale bars represent 200 μm.

To evaluate the surface modification efficacy in C3H10T1/2 cells by the ABC method, GFP-modified C3H10T1/2 cells and GFP-transfected C3H10T1/2 cells were analysed on a flow cytometer and examined by confocal microscopy. Figure 5A shows the dot plots of the respective groups (gating on GFP-positive cells: red dots). According to the analysis of dot plots, the percentages of GFP-positive cells in the control group, among GFP-transfected C3H10T1/2 cells, and among GFP-modified C3H10T1/2 cells were 0%, 7.3%, and 95.3%, respectively (Table 1). In accordance with the results of a flow cytometric analysis, most cells showed uniform fluorescence in the GFP-modified C3H10T1/2 cell group, whereas some of the cells showed sparse fluorescence in the GFP-transfected C3H10T1/2 cell group (Fig. 5B).

**Table 1:** The ratio of GFP-positive cells according to flow cytometric analysis.

	C3H10T1/2 (control)	GFP-transfected C3H10T1/2	GFP-modified C3H10T1/2
Ratio of GFP-positive cells	0% ± 0%	7.3% ± 1.2%*	95.3% ± 2.0%*

*p < 0.05 a statistically significant difference compared with the control group.

### *In vivo* duration of surface modification of cells

**Figure 6:**
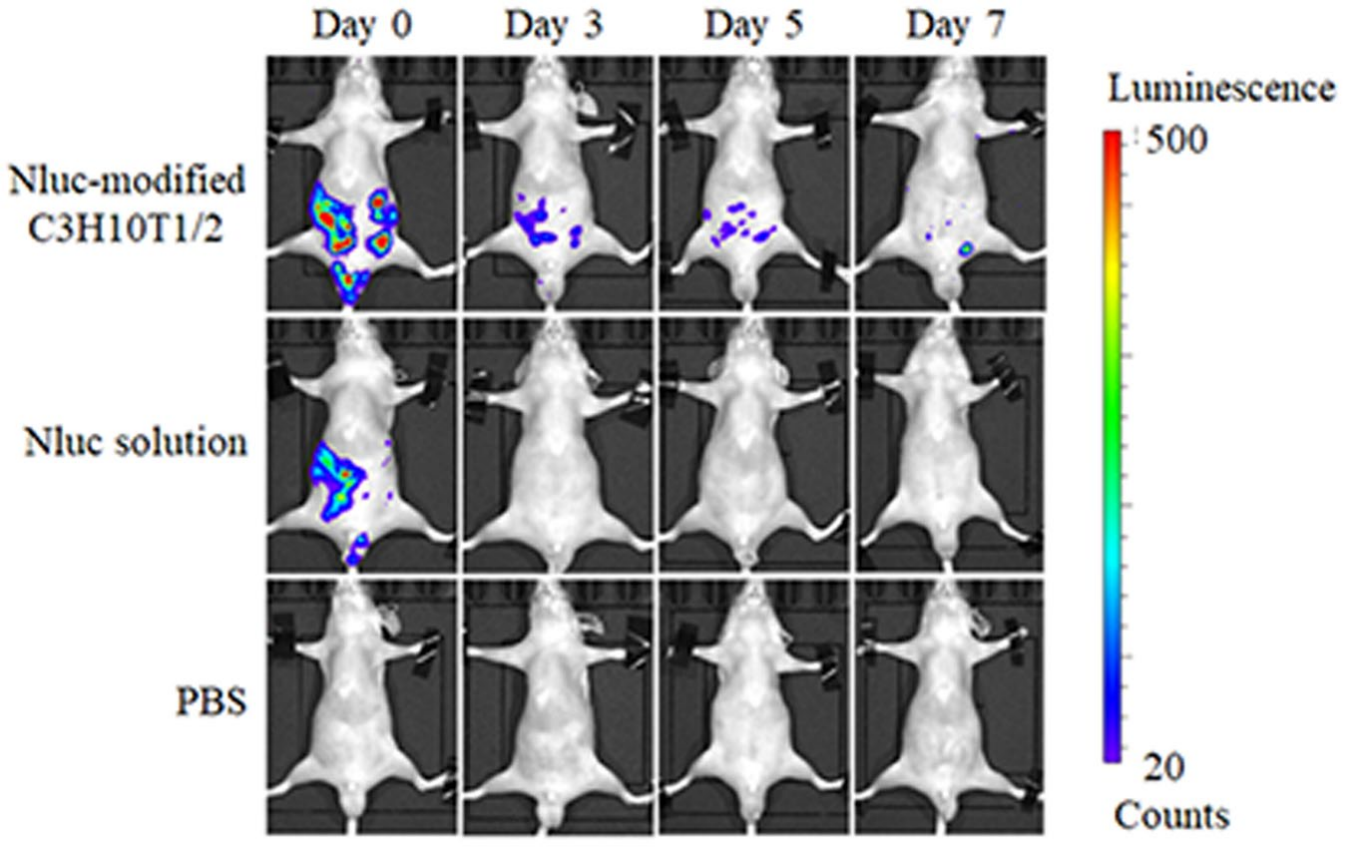
*In vivo* imaging of Nluc-modified C3H10T1/2 cells. The *in vivo* imaging of Nluc-modified C3H10T1/2 cells and an Nluc solution in nude mice by means of an IVIS imaging system. These photos were taken immediately (day 0), and 3, 5, and 7 days after administration. The experiments were conducted in duplicate, and a representative experiment is shown.

Finally, Nluc-modified C3H10T1/2 cells were intraperitoneally transplanted into mice, and the luminescence of cells in mice was traced by the in *vivo* imaging system (Fig. 6). The luminescence in Nluc-modified C3H10T1/2 cell-transplanted mice was the strongest immediately after the administration (day 0), and the luminescence decreased with time. However, the luminescence persisted for at least 7 days after the cell transplant. On the other hand, the luminescence in Nluc solution-injected mice was observed only on day 0 and was not detected on day 3 or later. As a control, any mice in the PBS group had no signals throughout the experimental period.

## Discussion

Cell functionalisation by surface modification methods is one of the most useful approaches to improving the therapeutic potential of cell transplantation. Some researchers focused on immune cells or the inflammatory reaction as the reason of the low viability of cells after transplantation, and PEGylation or heparinisation of cells by cell surface modification methods improves the therapeutic effectiveness of cells^27,28^. Although these methods functionalise cells transiently and maintain the *in vivo* presence of the cells, the duration of functionalisation (cell surface modification of chemicals) has been unclear. In some reports, the fluorescent materials modified to cells by various surface modification methods have been reported to disappear entirely within a few days after modification^20^. As a possible reason, the instability of chemicals may be due to the ineffective procedure for modification of cell surface, and optimal surface modification for the respective cell type has not been performed.

Click chemistry is one of the covalent modification methods, which makes the specific binding through the copper-catalyzed azide-alkyne cycloaddition under physiological conditions^29^. In the classical click chemistry for cell surface modification, it was concerned about the cytotoxicity of the copper-induced reactive oxygen species^30^. Recently, a copper-free click chemistry through the regioisomeric mixture of triazoles formed by the reaction of cyclooctynes and azides has been developed, and this was reported to hardly cause cytotoxicity because of the copper-free reaction^31,32^. Therefore, the copper-free click chemistry is widely used in biomedical fields and also applied to the cell surface engineering for various cells including MSCs^33^. However, the reaction time of the copper-free click chemistry requires over 60 min in PBS for appropriate modification, suggesting the complexity of this method. On the other hand, the ABC method completes the reaction in a short time of 5 min^34^. The quick reaction is one of the crucial advantages for the ABC method in the cell surface engineering.

In this study, we evaluated the duration of proteins modified to murine mesenchymal stem cells (C3H10T1/2 cells): a stable reporter protein (Nluc) as a model drug. We found that the ABC method ensures long-term modification to the cell surface *in vitro* and *in vivo* (Figs 2C and 6). Furthermore, the results in this study showed that the ABC method hardly affected the viability, proliferation and specific properties (migration ability and differentiation ability) of MSCs (Figs 2–4). Thus, the ABC method was confirmed to be an excellent cell surface modification procedure for MSCs.

In some studies using the ABC method, streptavidin is widely used for binding to biotin^35,36^. The advantages of streptavidin are greater stability, less non-specific adsorption, and higher solubility in comparison with avidin. The most important problem of avidin in terms of cell surface modification is the non-specific adsorption of various substances. In contrast, our results showed the lesser non-specific adsorption of drugs in the ABC method involving avidin (Figs 1 and 2C). The amount of drugs attached to cells depends on the concentration of sulfo-NHS (N-Hydroxysuccinimide)-LC-biotin (Fig. 2B); therefore, avidin, streptavidin, and their derivatives (i.e. NeutrAvidin, monomeric streptavidin) may be similarly suitable for the ABC method, respectively. In fact, few previous reports compared these compounds in terms of cell surface modification, and the details are needed to identify the better modification procedure.

The endocytosis of substances attached to cell surface is an important factor for drug modification of cells. Teramura *et al*. reported endocytosis of chemicals modified by various cell surface modification methods, and fluorescently labelling PEG-NHS, one of the covalent-bond methods, hardly caused the cytoplasmic uptake after modification^15,20^. In contrast, fluorescently labelling poly(vinyl alcohol)-alkyl, one of the hydrophobic interaction methods, showed uptake by cells after modification and return to the cell surface or exclusion from the cell surface afterwards^20,37^. Because our preliminary experiments revealed slight endocytosis of fluorescent drugs near the cell membrane at 2 h after modification by the ABC method, some percentage of the modified substances may be taken up by cells in some way. Furthermore, most substances taken up by cells are generally degraded in lysosomes within a short period. Our results showed that the amount of Nluc attached to cells stayed at approximately 80% on day 5 after the modification (Fig. 2C) and suggested that most of Nluc that is located on the cell surface was not degraded by lysosomes later. Further research is therefore needed for the detailed analysis of the substances modified to cells by the ABC method.

In the *in vivo* experiments, the apparently diminished luminescence in mice injected with Nluc-modified C3H10T1/2 cells on day 3 may be due to the cell death after the transplant (Fig. 6). This is because most cells generally disappear after a transplant by immunological factors^38,39^, a form of detachment-induced apoptosis^40,41^ and/or shear stress^42^ and some studies reported transplanted MSCs rapidly disappeared within a few days^43–46^. In Fig. 6, it is presumed that the greater number of Nluc-modified C3H10T1/2 cells disappeared in few days after transplantation because of these reasons. On the other hand, luminescence of Nluc-modified C3H10T1/2 cells was detected for at least 7 days, suggesting that Nluc remained on the surface of the cells for the period.

In conclusion, MSCs were easily and rapidly functionalised by the ABC method without major effects on cell characteristics (viability, proliferation, migration, and differentiation) and the duration of surface modification with drugs was relatively long *in vitro* and *in vivo*, suggesting that the ABC method may be useful for an effective MSC-based therapy.

## Methods

### Materials

Dulbecco’s modified Eagle’s medium (DMEM), a trypsin-EDTA solution (0.25% trypsin and 1 mM EDTA), and Fluoro-KEEPER Antifade Reagent were purchased from Nacalai Tesque Inc. (Kyoto, Japan). Hanks’ balanced salt solution (HBSS), Alizarin Red S (3,4-Dihydroxy-9,10-dioxo-2-anthracenesulfonic acid sodium salt), and Oil Red O (1-(2,5-dimethyl-4-(2,5-dimethylphenyl) phenyldiazenyl) azonapthalen-2-ol) were purchased from Sigma-Aldrich Co. (St. Louis, MO, USA). Sulfo-NHS-LC-biotin (sulfosuccinimidyl-6-[biotin-amido]hexanoate) was acquired from Pierce Chemical Co. (Rockford, IL, USA). Isoflurane, avidin (from egg white), 25% glutaraldehyde, and a 0.4% trypan blue solution were purchased from Wako Pure Chemical Industries, Ltd. (Osaka, Japan). Cell Counting Kit-8 (CCK-8) was obtained from Dojindo Laboratories (Kumamoto, Japan). All other chemicals were of the highest grade commercially available.

### Animals

Male nude BALB/c Slc-*nu*/*nu* mice (5-week-old) were purchased from Japan SLC, Inc. (Shizuoka, Japan). The mice were maintained under specific pathogen-free (SPF) conditions. All the animal experiments were conducted in accordance with the principles and procedures outlined in the National Instituted of Health Guide for the Care and Use of Laboratory Animals. The protocols for animal experiments were approved by the Animals Experimentation Committee of Kyoto Pharmaceutical University.

### Cell culture

A murine mesenchymal stem cell line (C3H10T1/2 cells) was obtained from Dr. Hiroki Kagawa (Department of Cell Biology, Kyoto Pharmaceutical University, Kyoto, Japan) and cultured in DMEM supplemented with 15% of heat-inactivated foetal bovine serum and 0.6% of an antibiotic-antimycotic mixed solution. A murine colon carcinoma cell line stably expressing the firefly luciferase gene (colon26/luc cells) was obtained from Dr. Makiya Nishikawa (Department of Biopharmaceutics and Drug Metabolism, Graduate School of Pharmaceutical Sciences, Kyoto University, Kyoto, Japan) and cultured in DMEM supplemented with 10% of heat-inactivated foetal bovine serum and 0.6% of an antibiotic-antimycotic mixed solution. These cells were maintained in a humidified atmosphere containing 5% of CO_2_ at 37 °C (in an incubator).

### Purification and biotinylation of reporter proteins

The extraction of Nluc and GFP was performed by the affinity purification method of His-tagged fusion proteins. Briefly, the *His-Nluc* gene was polymerase chain reaction (PCR)-amplified from the pNL2.3 [secNluc/Hygro] vector (Promega Co., Madison, WI, USA) with specific primers (forward: 5′-CTTGAATTCATGGTCTTCACACTCGAAGAT-3′, reverse: 5′-TCTGGATCCTTACGCCAGAATGCG-3′) using DNA polymerase KOD -Plus- Ver. 2 (Toyobo Co., Ltd., Osaka, Japan). Similarly, the *His-GFP* gene was PCR-amplified from the pCMV-GFP vector (Addgene plasmid # 11150) with specific primers (forward: 5′-CTTGAATTCATGCATCACCATCACCATCACGTGAGCAAGG-3′, reverse: 5′-TCTGGATCCTTACTTGTACAGCTC-3′). The DNA fragment coding His-Nluc and His-GFP was inserted into the pFN18A vector (Promega Co., Madison, WI, USA) digested with *EcoR*I and *BamH*I with Ligation high Ver. 2 (Toyobo Co., Ltd., Osaka, Japan), resulting in pFN18A-His-Nluc and pFN18A-His-GFP. Subsequently, *E. coli* KRX competent cells (Promega) were transformed by heat shock with the plasmids and incubated in the Luria-Bertani (LB) medium at 37 °C for 12 h. After that, expression of each protein was induced by adding 0.01% L-rhamnose with incubation at room temperature for 12 h. The cells were lysed and the protein from the lysate was purified using the His60 Ni Superflow Resin (Clontech Laboratories, Inc., Mountain View, CA, USA). For the preparation of biotinylated His-Nluc (biotin-Nluc) or biotinylated His-GFP (biotin-GFP), His-Nluc or His-GFP was dissolved in 0.1 M phosphate buffer (pH 8.0) to attain a concentration of 1 mg/mL. His-Nluc or His-GFP solution was mixed with 1.6 mM sulfo-NHS-LC-biotin and incubated at room temperature for 30 min. The reaction solution was centrifuged at 4,000 × *g* for 5 min using a Vivaspin 20 concentrator (10,000 molecular weight cut-off, Sartorius AG, Goettingen, Germany) and a 100 μM biotin-Nluc or biotin-GFP solution was prepared in HBSS.

### Surface modification of cells by the ABC method

C3H10T1/2 cells (2 × 10^5^ cells/well) were seeded in 6-well culture plates and incubated at 37 °C in a humidified 5% CO_2_ incubator overnight. C3H10T1/2 cells were then incubated with 50, 100, 500, 1000, 5000, or 10000 μM sulfo-NHS-LC-biotin dissolved in HBSS for 10 min at room temperature. Biotinylated C3H10T1/2 cells were washed twice with PBS and incubated with 100 μg/mL avidin dissolved in HBSS for 5 min. Finally, avidinated C3H10T1/2 cells were washed twice with PBS and incubated with 100 μM biotin-Nluc for 5 min. Nluc-modified C3H10T1/2 cells were washed with PBS and incubated in the medium at 37 °C in a humidified 5% CO_2_ incubator. The amount of drugs modified to the cell surface was evaluated by measuring the luciferase activity in lysed cells using the Nano-Glo Luciferase Assay Reagent (Promega) after detaching cells with cell scrapers (IWAKI Co., Kyoto, Japan). In addition, the viability of cells was measured by means of the CCK-8 kit 24 h after seeding of the surface-modified cells in a 96-well plate. Separately, C3H10T1/2 cells (2 × 10^5^ cells/well) were seeded in 6-well culture plates and incubated at 37 °C in a humidified 5% CO_2_ incubator overnight. C3H10T1/2 cells were then incubated with 500 μM NHS-FITC (Thermo Fisher Scientific, Inc., Waltham, MA, USA) dissolved in PBS for 10 or 20 min at room temperature. After washing twice with PBS, FITC-modified C3H10T1/2 cells were detached with cell scrapers and seeded to a 96-well plate (1.5 × 10^4^ cells /well). The fluorescence of FITC-modified C3H10T1/2 cells was detected using a multi-label plate reader (ARVO MX Wallac 1420 Multilabel Counter, PerkinElmer, Wellesley, MA, USA).

### Confocal microscopic examination of surface-modified cells

GFP-modified C3H10T1/2 cells and C3H10T1/2 cells (control) were seeded to a Lab-Tek II chamber slide (Nunc, Rochester, NY) and incubated for 3 h at 37 °C in a humidified 5% CO_2_ incubator and fixed with a 2% glutaraldehyde solution for 2 h at 4 °C. GFP-modified C3H10T1/2 cells were prepared by adding 100 μM biotin-GFP after addition of 100 μg/mL avidin to biotinylated C3H10T1/2 cells. C3H10T1/2 cells (control) were prepared by adding biotin-GFP to unmodified C3H10T1/2 cells after addition of avidin. To prepare the fluorescent streptavidin-modified C3H10T1/2 cells, biotinylated C3H10T1/2 cells were seeded to a Lab-Tek II chamber slide and incubated for 3 h at 37 °C in a humidified 5% CO_2_ incubator and fixed with a 2% glutaraldehyde solution for 2 h at 4 °C, and then 33 μg/mL Alexa fluor 647 conjugate of streptavidin (Biotium, Hayward, CA) was added to biotinylated C3H10T1/2 cells after removal of the glutaraldehyde solution. After washing with PBS and mounting with Fluoro-KEEPER Antifade Reagent, we examined fluorescently labelled C3H10T1/2 cells or GFP-modified C3H10T1/2 cells under an A1R confocal laser scanning microscope (Nikon, Tokyo, Japan). To evaluate the surface modification efficacy in C3H10T1/2 cells by the ABC method, GFP-modified C3H10T1/2 cells and GFP-transfected C3H10T1/2 cells were seeded to a Lab-Tek II chamber slide (Nunc, Rochester, NY) and incubated overnight in a humidified 5% CO_2_ incubator and fixed with a 2% glutaraldehyde solution for 2 h at 4 °C. After washing with PBS and mounting with Fluoro-KEEPER Antifade Reagent, we observed these cells under an A1R confocal laser scanning microscope (Nikon, Tokyo, Japan).

### Evaluation of the cell proliferation, attachment, and migration

Nluc-modified C3H10T1/2 cells (with 1000 μM sulfo-NHS-LC-biotin) were prepared as described above. To evaluate the cell proliferation, Nluc-modified C3H10T1/2 cells and untreated C3H10T1/2 cells (2 × 10^5^ cells/well) were incubated in a 6-well plate and detached with cell scrapers every day starting on the next day. The number of cells was evaluated by a 0.4% trypan blue exclusion assay, and the PDT of cells was calculated by means of the following equation: PDT = T × ln2 ÷ ln(N_t_/N_i_), where T is incubation time (h), N_t_ is the cell number at time t, and N_i_ is the initial cell number (at time zero). To evaluate the cell attachment, Nluc-modified C3H10T1/2 cells and untreated C3H10T1/2 cells (2 × 10^5^ cells/well) were seeded in a 96-well plate and incubated for 10, 30, or 90 min. After that, the cells were washed with PBS twice, and the number of cells attached to the plate was evaluated by the 0.4% trypan blue exclusion assay. In addition, to evaluate the cell attachment at 24 h, Nluc-modified C3H10T1/2 cells and untreated C3H10T1/2 cells (1.5 × 10^4^ cells/well) were seeded in a 96-well plate and incubated for 24 h. After that, the cells were washed with PBS and the number of cells attached to the plate was evaluated by using CCK-8 kit. To evaluate the cell migration, Nluc-modified C3H10T1/2 cells and untreated C3H10T1/2 cells (4 × 10^4^ cells/well) were seeded in a 24-well plate containing Transwell permeable supports (inserts) with 8.0-μm membrane pore size (Corning Inc., Corning, NY, USA) in the serum-free medium (upper well). These cells were incubated for 24 h after addition of the serum-free medium or for 48 h in the conditioned medium from colon26/luc cells in the bottom well. These cells were then fixed with a 2.5% glutaraldehyde solution for 2 h at 4 °C and the upper cells were removed by a piece of cotton. The remaining cells (basal side in the upper well) were stained with crystal violet and the number of cells was determined by examination under a digital microscope (BZ-8000, Keyence, Osaka, Japan).

### Cell differentiation

To evaluate the differentiation potential, Nluc-modified C3H10T1/2 cells were induced to differentiate into adipocytes, osteoblasts and chondrocytes according to the instruction manual of Mesenchymal Stem Cell Media (Promocell GmbH, Heidelberg, Germany) by referring to previous reports^47–49^ (Fig. 4).

To differentiate into adipocytes, Nluc-modified C3H10T1/2 cells and untreated C3H10T1/2 cells (5 × 10^3^ cells/well) were seeded to a 96-well plate and incubated in normal medium for 48 h. Confluent cells were incubated in the mesenchymal stem cell adipogenic differentiation medium (Promocell GmbH, Heidelberg, Germany) for 14 days and the medium was refreshed every third day. The cells induced to differentiate into adipocytes were stained using Oil Red O to detect adipocytes. Similarly, for differentiation into osteoblasts, the cells were seeded in a 96-well plate and incubated in a normal medium for 72 h. Subconfluent cells were incubated in the mesenchymal stem cell osteogenic differentiation medium (Promocell GmbH, Heidelberg, Germany) for 21 days and the medium was refreshed every third day. Cells induced to differentiate into osteoblasts were stained using Alizarin Red S to detect osteoblasts. These cells were examined under a BZ-8000 digital microscope. For differentiation into chondrocytes, the cells (2.5 × 10^5^ cells/well) were seeded to a 96-well U-bottom plate and incubated in a normal medium until the cells form a spheroid shape. Spheroids were incubated in the mesenchymal stem cell chondrogenic differentiation medium (Promocell GmbH, Heidelberg, Germany) for 21 days and the medium was refreshed every third day. Then, the cells were stained using Alcian Blue staining solution (Merck, Darmstadt, Germany), and examined under a BZ-9000 digital microscope (Keyence, Osaka, Japan).

### Flow cytometric analysis

GFP-modified C3H10T1/2 cells (2 × 10^5^ cells/well) prepared as described above were seeded in a 6-well plate. Separately, C3H10T1/2 cells (10^5^ cells/well) were seeded in a 24-well plate, transfected with a pCMV-GFP plasmid using Lipofectamine 3000 (Invitrogen Life Technologies Inc., Carlsbad, CA, USA). In brief, a pCMV-GFP (1 µg) and P3000 reagent (2 μL, Invitrogen Life Technologies Inc.) were mixed in Opti-MEM (50 μL). Then, the premixed DNA and Lipofectamine 3000 (3 μL) were mixed in Opti-MEM (50 μL) and incubated for 5 min at room temperature. DNA-lipid complex was added to cells. After 24 h, the medium was exchanged to the culture medium (DMEM supplemented with 15% of heat-inactivated fetal bovine serum and 0.6% of an antibiotic-antimycotic mixed solution.) and incubated for additional 24 h. These cells were detached using cell scrapers, and then the suspended cells were fixed with a 2.5% glutaraldehyde solution for 2 h at 4 °C and kept on ice until analysis. These cells were analysed on BD LSRFortessa flow cytometer (Becton Dickinson, Sunnyvale, CA, USA) and data processing was conducted in the FACS Diva software. The confocal microscopic examination of GFP-transfected cells or GFP-modified cells was carried out as described above.

### An *in vivo* imaging assay after cell transplantation

Nluc-modified C3H10T1/2 cells (10^6^ cells per 100 μL) or a Nluc solution (3 ng/mL) were intraperitoneally injected into Balb/c Slc-*nu/nu* mice under isoflurane anaesthesia. Then, 100 μL of luciferin solution (Nano-Glo Luciferase Assay Substrate diluted with PBS to final concentration of 1%) was intraperitoneally administered to the mice immediately, and 3, 5, and 7 days after the administration and the luminescence in the respective mice was detected and imaged on an IVIS imaging system (Xenogen, Alameda, CA).

### Statistical analysis

Statistical significance of differences was evaluated by one-way analysis of variance (ANOVA) followed by Dunnett’s test for multiple comparisons (versus a control), Tukey’s test or by Student’s *t* test for two groups. Difference with P values less than 0.05 were: considered statistically significant.

### Data availability statement

All data used for this research are publicly available.

## Electronic supplementary material

Supplementary information
