# Supplementary material for: Long-term drug modification to the surface of mesenchymal stem cells by the avidin-biotin complex method
Source: Sci Rep. 2017 Dec 5;7:16953. doi: 10.1038/s41598-017-17166-8 (PMC5717103; doi:10.1038/s41598-017-17166-8)
Supplement: Supplementary file 1 — Supplementary information [file 41598_2017_17166_MOESM1_ESM.pdf]

Supplementary information

Long-term drug modification to the surface of mesenchymal stem cells by the avidin-biotin complex method

Yukiya Takayama, Kosuke Kusamori, Mika Hayashi, Noriko Tanabe, Satoru Matsuura, Mari Tsujimura, Hidemasa Katsumi, Toshiyasu Sakane, Makiya Nishikawa and Akira Yamamoto

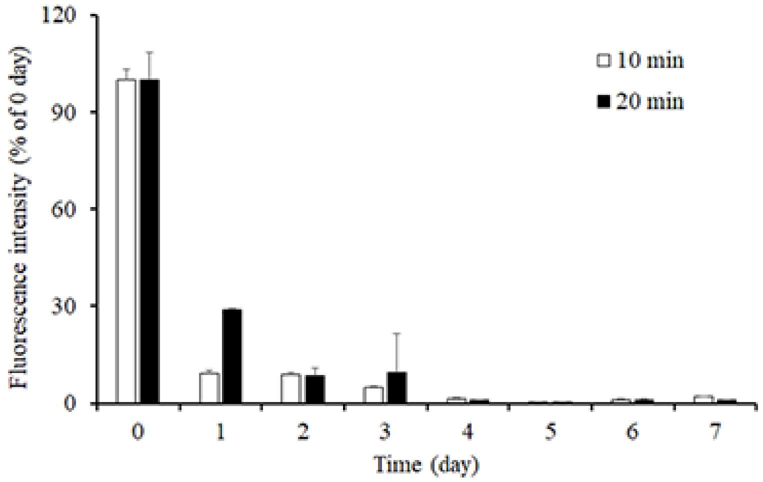

Supplementary Fig. 1. The fluorescence intensity of FITC-modified C3H10T1/2 cells prepared using 500 μM NHS-FITC. C3H10T1/2 cells were incubated with NHS-FITC for 10 min (white) and 20 min (black). Results are expressed as means ± SD (n = 4).

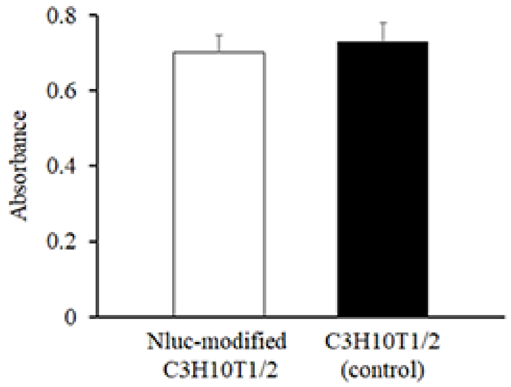

Supplementary Fig. 2. Adhesion of Nluc-modified C3H10T1/2 cells onto cell culture plate at 24 h. Results are expressed as means ± SD (n = 4).

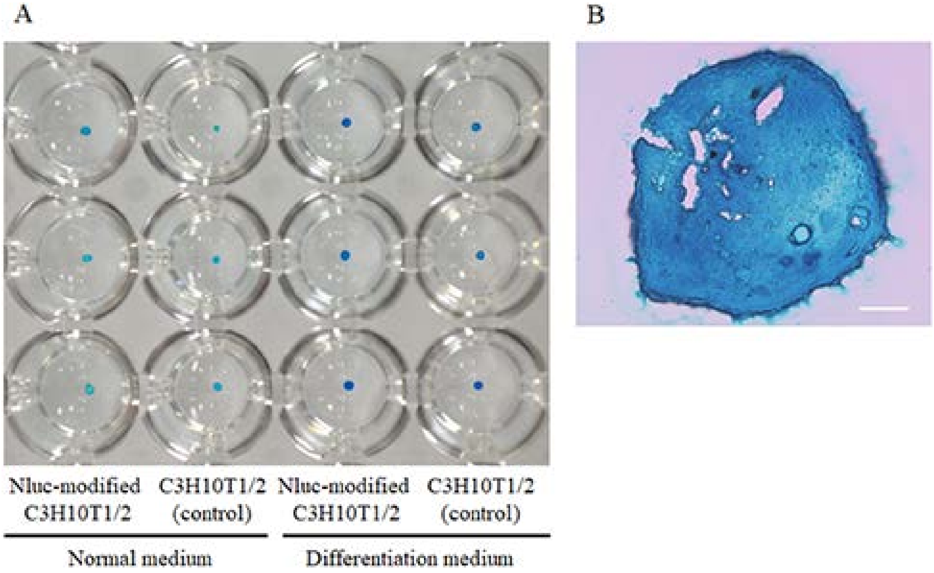

Supplementary Fig. 3. Differentiation of Nluc-modified C3H10T1/2 cells into chondrocytes. (A) The typical images of Nluc-modified C3H10T1/2 cells and C3H10T1/2 cells (control) that differentiated into chondrocytes. (B) The cryosection image of Nluc-modified C3H10T1/2 cells after Alcian Blue staining. Scale bar represents 100 μm.
